# OD16 Assessing Technical Aids In Europe: Are There Any Specific Health Technology Assessment Methods To Assess Technical Aids?

**DOI:** 10.1017/S0266462324001508

**Published:** 2025-01-07

**Authors:** Julie Bonamour, Camille Marguerite, Feyriel Bouaraba, Judith Fernandez, Hubert Galmiche

## Abstract

**Introduction:**

Technical aids (TAs) are health devices (e.g., hearing aids, prostheses, wheelchairs) used to restore a body function or compensate for disability. In France, TAs are assessed based on clinical data, as are any medical devices (MDs). Considering that TAs are special MDs (no curative action), specific assessment methods might be proposed. The methods used in Europe were explored.

**Methods:**

To learn about TA evaluation practices in Europe, the French National Authority for Health (HAS) sent a survey to nine identified European health technology assessment (HTA) agencies. The questionnaire was specific to TAs, and the questions asked were about: (i) the reimbursement of TAs (reimbursed or not, at national or local level), (ii) the HTA process (HTA process or not, HTA process description), and (iii) the methods used (any specific methods for assessing TAs other than HTA methods to assess “classic” MDs).

**Results:**

All the European HTA agencies contacted provided a response. All these countries have a healthcare system that allows TAs to be reimbursed. In Austria, Ireland, Italy, the Netherlands, Norway, Portugal, Spain, and the United Kingdom, TAs must be CE-marked and comply with technical standards to be reimbursed; no clinical data are required. However, the National Institute for Health and Care Excellence provides recommendations for certain TA categories. In Germany, the CE-marking and the respect of technical standards are mandatory, and certain TAs need to show a medical benefit (e.g., orthoses); nevertheless, there are no specific methods to assess TAs.

**Conclusions:**

Overall, the feedback obtained does not reveal any specific evaluation method applied to TA assessment in the various European countries analyzed. France stands out from these European countries regarding the assessment of TAs because of its one-stop-shop system for health technology developers and the HAS requirements for technical and clinical data.

